# Leveraging machine learning and rule extraction for enhanced transparency in emergency department length of stay prediction

**DOI:** 10.3389/fdgth.2024.1498939

**Published:** 2025-02-12

**Authors:** Waqar A. Sulaiman, Charithea Stylianides, Andria Nikolaou, Zinonas Antoniou, Ioannis Constantinou, Lakis Palazis, Anna Vavlitou, Theodoros Kyprianou, Efthyvoulos Kyriacou, Antonis Kakas, Marios S. Pattichis, Andreas S. Panayides, Constantinos S. Pattichis

**Affiliations:** ^1^ Department of Computer Science and Biomedical Engineering Research Centre, University of Cyprus, Nicosia, Cyprus; ^2^ CYENS Centre of Excellence, Nicosia, Cyprus; ^3^ Research & Development Department, 3AHealth, Nicosia, Cyprus; ^4^ Department of Intensive Care Medicine, Limassol General Hospital, State Health Services Organisation, Nicosia, Cyprus; ^5^ Department of Critical Care and Emergency Medicine, Medical School, University of Nicosia, Nicosia, Cyprus; ^6^ Department of Critical Care, St Thomas's Hospital NHS, London, United Kingdom; ^7^ Department of Electrical Engineering, Computer Engineering and Informatics, Cyprus University of Technology, Limassol, Cyprus; ^8^ Department of Electrical and Computer Engineering, University of New Mexico, Albuquerque, NM, United States

**Keywords:** emergency department, length of stay, machine learning, gradient boosting, rule extraction, predictive modeling, explainable AI, healthcare analytics

## Abstract

This study aims to address the critical issue of emergency department (ED) overcrowding, which negatively affects patient outcomes, wait times, and resource efficiency. Accurate prediction of ED length of stay (LOS) can streamline operations and improve care delivery. We utilized the MIMIC IV-ED dataset, comprising over 400,000 patient records, to classify ED LOS into short (≤4.5 hours) and long (>4.5 hours) categories. Using machine learning models, including Gradient Boosting (GB), Random Forest (RF), Logistic Regression (LR), and Multilayer Perceptron (MLP), we identified GB as the best performing model outperforming the other models with an AUC of 0.730, accuracy of 69.93%, sensitivity of 88.20%, and specificity of 40.95% on the original dataset. In the balanced dataset, GB had an AUC of 0.729, accuracy of 68.86%, sensitivity of 75.39%, and specificity of 58.59%. To enhance interpretability, a novel rule extraction method for GB model was implemented using relevant important predictors, such as triage acuity, comorbidity scores, and arrival methods. By combining predictive analytics with interpretable rule-based methods, this research provides actionable insights for optimizing patient flow and resource allocation. The findings highlight the importance of transparency in machine learning applications for healthcare, paving the way for future improvements in model performance and clinical adoption.

## 1 Introduction

Emergency departments (EDs) are at the forefront of healthcare, where providing timely and efficient care is essential for saving lives. Overcrowding is a global problem that causes longer wait times, lower quality care, and increased stress for healthcare providers (1). Overcrowding occurs when the demand for emergency services exceeds a department’s capacity to provide timely, high-quality care, negatively affecting patient outcomes and operational efficiency (2).

Effective management of emergency department (ED) length of stay (LOS) is crucial to mitigating overcrowding, primarily by optimizing the allocation of resource (3). Accurate prediction of LOS can help streamline patient flow, prioritize treatment, and ensure that resources are used efficiently, resulting in better patient outcomes in the ED. Automating ED LOS classification with machine learning (ML) techniques allows healthcare providers to make proactive data-driven decisions, especially in high-volume EDs where traditional methods are not scalable or accurate (4).

Traditional LOS prediction methods often use simplistic metrics or heuristic rules, which do not account for the complexity of patient conditions and clinical pathways (4). These approaches introduce biases and overlook important patient-specific factors, reducing resource planning effectiveness and increasing the likelihood of suboptimal care delivery, particularly under high-pressure conditions typical of EDs.

Recent advances in machine learning have shown promise in improving the accuracy and granularity of ED LOS classification (5, 6). However, many ML models are considered black boxes, which limits their practical application in clinical settings due to difficulties in understanding the underlying decision processes (7–9). To address this issue, our study aims not only to accurately classify ED LOS using the MIMIC IV ED healthcare dataset, but also to improve model interpretability by implementing a novel rule extraction method tailored for Gradient Boosting (GB).

We use machine learning and deep learning models such as GB, Random Forest (RF), Logistic Regression (LR), and Multilayer Preceptron (MLP) to categorize ED LOS into short stays (≤4.5 h) and long stays (>4.5 h), identifying the model with the best predictive capabilities. In addition, our goal is to improve model transparency by analyzing the extracted rules and calculating metrics such as accuracy, relative coverage, and overall coverage. This rigorous analysis provides healthcare professionals with clear and actionable guidelines for patient management, facilitating informed clinical decision-making, and optimal allocation of resources in emergency departments (10, 11).

Our research also addresses the class imbalance of the data set, which shows that “ED LOS (Long)” cases outnumber “ED LOS (Short)” cases. To address this imbalance, we employ various different types of over- and under-sampling methods in a robust manner, enhancing the model’s ability to learn from both classes effectively (12).

By validating these findings and exploring the integration of sophisticated predictive models with interpretable rule extraction techniques, this research aims to optimize patient flow and refine care delivery strategies in emergency healthcare settings. This approach contributes to the advancement of data-driven methodologies in healthcare management, illustrating how ML techniques can enhance operational efficiency and patient outcomes.

**Figure 1:**
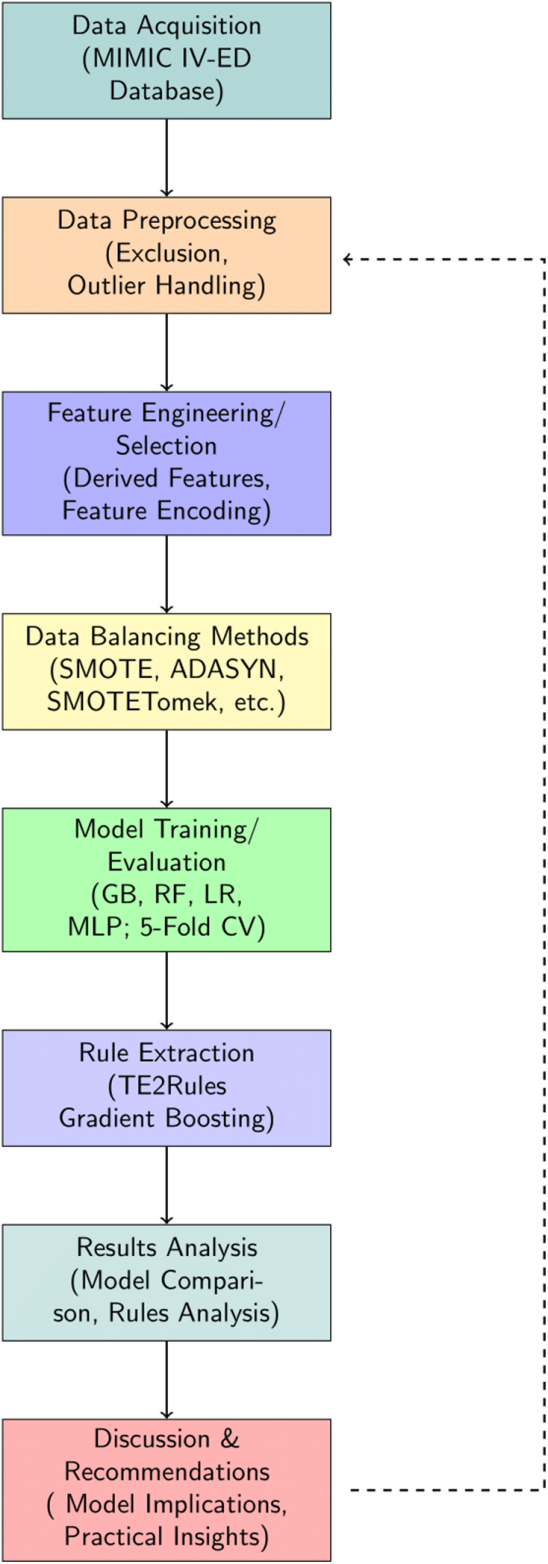
Overview of study workflow.

The structure of the paper is organized as follows: Section 2 provides an overview of studies on Emergency Department Length of Stay. Section 3 details the materials and methods, including feature selection, machine learning modeling, and rule extraction. Section 4 presents the results of the feature analysis, model evaluation, and rule extraction. Section 5 discusses these findings, and finally Section 6 concludes the article. A complete overview of the study flow is presented in Figure 1.

## 2 An overview of emergency department length of stay studies

Recent investigations into predicting emergency department length of stay have utilized various machine learning models to meet the growing demand for effective patient care and resource management. The objectives, features, datasets, and methodologies of these studies vary, as shown in Table 1. This section provides a comprehensive overview of current research in this area.

**Table 1:** Summary of studies on ED LOS prediction.

Study	Objective	Features	Dataset	Models	Sample size and train/test split	Results
Ala et al. (13)	Classify emergency patients into complex and fast-track (<4 h)	19 variables which include vital signs, pain, chief complaints, arrival means, and age	MIMIC-IV-ED and MIMIC-IV	MLP, CART, RF, LR, SGB	sample size = 104,014, Split: 80% train, 20% test, 5-folds CV	SGB: Sensitivity = 59.13%, Specificity = 91.20%, AUC = 0.752
Zebin et al. (14)	Identify short (0–7 d) and long (>7 d) hospital stays	Admission records, demographics, diagnosis codes (ICD-9), chart events	MIMIC-III	RF, Auto-Encoder, DNN	Sample Size: 53,104 Split: 80% train, 10% validation, 10% test	Auto+DNN: Accuracy = 77.7%, Sensitivity = 77.6%, Precision = 75.2%
Pedro et al. (15)	Predict hospital admission and prolonged LOS in older adults (>70 years)	21 variables including age, sex, comorbidities, ISAR tool, FRAIL scale, and CAM	Retrospective cohort of geriatric ED in Brazil	PRO-AGE scoring system, Bootstrapping	Sample Size: 5,025 patients, Data Split: 2:1 (bootstrapped)	AUC = 0.79
Bopche et al. (16)	Predict prolonged ED LOS (>2 d) using historical patient records	Age, temporal features, laboratory results, diagnostic codes	Retrospective cohort at St Olavs University Hospital, Norway	XGBoost, LightGBM, CatBoost	Sample Size: 35,591 Split: 80/20	XGBoost: AUROC = 0.9797, Accuracy = 0.9286, Sensitivity = 0.9179, Specificty = 0.9322
Etu et al. (17)	Prediction model for COVID-19 patient ED LOS less than or greater than 4 h	127 clinical and operational variables including age, sex, ESI, insurance, and comorbidities	Henry Ford Hospital EHR	LR, GB, DT, RF,	Sample Size: 57,665 Split: 80% train, 20% test, SMOTE, 10-fold cross-validation	GB: Accuracy = 85%, AUC = 0.930, F1 Score = 0.880,
Chang et al. (18)	Predict low-severity patients with short discharge LOS in ED (<4 h)	32 variables including trauma, chief complaints, injury mechanisms, and vital signs	Retrospective study in Taiwan	CatBoost, XGBoost, DT, RF, LR	Sample Size = 127,749, Split: 80% train, 20% test	CatBoost: AUC = 0.748, Sensitivity = 59.09%, Specificity = 83.25%
Rahman et al. (19)	Predict ED LOS >4 h using a decision tree algorithm	33 variables including ED visit type, age, gender, triage category, and consultations	Regional Australian public hospital	Decision Tree	Sample Size: 80,512 Split: divided into mutually exclusive horizontal segments, 10-fold cross-validation	Accuracy = 84.94%
Gill et al. (20)	Predict fast track patients staying longer than 4 h in ED	27 variables with emphasis on time-dependent factors like time to imaging request	Regional Australian Public Hospital	Gradient Boosting	Sample Size: 62,955 Split: 70% train, 30% test, 10-fold cross-validation	AUC = 0.890
This study	Predict ED LOS >4.5 h with enhanced interpretability using rule extraction	Top 10 important variables including arrival methods and medication history	MIMIC IV-ED	GB, RF, LR, SVM,	Sample Size = 410,927, Split: 80% train, 20% test, SMOTE+Tomek, 5-fold stratified cross-validation	GB: Accuracy = 69.93%, Sensitivity = 88.20%, Specificity = 40.95%, AUC = 0.730

CatBoost, Categorical boosting; CART, Classification and regression trees; DNN, Deep neural network; DT, Decision tree; GB, Gradient boosting; LR, Logistic regression; MLP, Multilayer perceptron; ProAGE, Pronto atendimento geriátrico especializado-specialized geriatric emergency care; RF, Random forest; SGB, Stochastic gradient boosting; SMOTE + Tomek, Synthetic minority over-sampling technique + tomeklinks undersampling technique; XGBoost, Extreme gradient boosting.

Ala et al. (13) and Zebin et al. (14). Both used the MIMIC data set, although different versions, to investigate patient outcomes in emergency settings. Ala et al. (13) classified emergency patients into complex and fast-track categories using models such as Multilayer Perceptron (MLP), Regression Trees (CART), and Stochastic Gradient Boosting (SGB), with the SGB model reaching an AUC of 0.752. This study demonstrated the importance of clinical variables, such as vital signs and chief complaints, in designing successful decision support systems. Zebin et al (14) used an auto-encoded deep neural network on the MIMIC-III dataset to differentiate between short and long hospital stays. Their solution, which obtained an accuracy of 77.7%, demonstrated the value of advanced feature engineering and deep learning to capture complex patterns in large datasets. Both studies emphasize the need to integrate diverse patient data to improve prediction accuracy and clinical decision-making.

Pedro et al. (15) used a ProAGE (Pronto Atendimento Geritrico Especializado – Specialized Geriatric Emergency Care) rating system which is a validated mnemonic method used to assess vulnerability and predict hospital admission, prolonged length of stay (LoS), and death in older adults at the ED (21). They combined ProAGE with bootstrapping to predict hospital admission and prolonged LOS in older people, achieving an AUC of 0.790. Their study focused on a specific cohort, emphasizing the importance of geriatric-specific characteristics such as the ISAR (Identification of Seniors At Risk) tool and the FRAIL scale (Fatigue, Resistance, Ambulation, Illness, and Loss of weight), which are critical to understanding the complexities of care for elderly patients.

Etu et al. (17) created a prediction model for COVID-19 patient ED LOS by combining Logistic Regression (LR), Gradient Boosting (GB), and Decision Trees. Their study, which used data from Henry Ford Hospital, found that the GB models outperformed other models, with an AUC of 0.930, especially when dealing with unbalanced data sets using approaches such as SMOTE.

Chang et al. (18) used a variety of models, including CatBoost and XGBoost, to predict low-severity patients with short discharge times. Their work revealed the usefulness of ensemble methods in a retrospective Taiwanese data set, with CatBoost reaching an AUC of 0.748. This study emphasizes the necessity of model selection in different clinical contexts.

Rahman et al. (19) and Gill et al. (20) both studied regional Australian public hospitals, with an emphasis on predicting ED LOS of more than 4 h. Rahman et al. (19) used a decision tree approach and obtained an accuracy of 84.94%. Their research revealed the utility of decision trees in developing interpretable models for the prediction of LOS in the emergency department, which is especially significant in clinical settings where transparency is required. Similarly, Gill et al. (20) focused on fast-track patients and applied gradient boosting, reaching an AUC of 0.890. Their results highlighted the importance of time-dependent variables, such as the time to imaging request, in understanding patient flow and addressing bottlenecks in emergency care. Both findings emphasize the importance of predictive modeling in increasing the efficiency of the emergency department and patient outcomes.

Our research builds on previous work by combining rule extraction with GB models to improve interpretability while retaining predictive performance. Our research not only coincides with the accuracy and AUC scores published in previous studies but also makes a distinct addition by offering clear, actionable insights through rule extraction.

## 3 Materials and methods

In this section, we first describe raw data processing/ benchmark data generation, and preprocessing. Next, we introduce data balancing methods, baseline models for the benchmark task, and model performance evaluation. Finally, we elaborate on the rule extraction and analysis methods. A complete overview of the study flow is presented in Figure 1.

### 3.1 Data source

We used real-world data from the MIMIC-IV ED dataset (22), which is a comprehensive clinical database that contains detailed information from the emergency departments of Beth Israel Deaconess Medical Center in Boston, Massachusetts. The analytical data set contained deidentified information from more than 400,000 emergency department visits between 2011 and 2019, such as demographics, comorbidities, laboratory results, vital signs, medications, medical procedures, and clinical results. The raw data was managed using a data generation pipeline (7), and additional data processing was performed with Python version 3.9.7. Additional information about the initial data processing is provided to ensure the transparency and reproducibility of our methods.

### 3.2 Preprocessing

Raw electronic health record (EHR) data is often unsuitable for model building due to issues such as missing values, outliers, duplicates, and errors arising from system or clerical errors (23). Therefore, effective preprocessing is crucial to ensure optimal machine learning performance.

#### 3.2.1 Exclusion criteria

We addressed these issues by first defining the following exclusion criteria:

• Patients under the age of 18 (*n* = 168) were excluded
• ED LOS less than 0.5 h (*n* = 2,264) and more than 24 h (*n* = 11,896) were excluded.
• Negative values of ED LOS (*n* = 6) that were deemed erroneous were removed.

The criteria were guided by existing literature (7, 24), which shows that patients under the age of 18 should be discarded as their medical conditions and healthcare requirements differ significantly from adults, necessitating different triage and treatment procedures. An ED LOS of 24 to 48 h can be considered prolonged (24). Prolonged LOS is frequently attributed to non-health-related factors such as bed shortages or patients refusing to be discharged, whereas stays of less than 0.5 h are typically associated with minor complications.

#### 3.2.2 Outlier detection

Outlier detection was performed, first by defining values of vital signs as outliers and marking them as missing if they fell outside the plausible physiological range determined by domain knowledge, such as a value less than zero or an oxygen saturation level greater than 100%, using an outlier detection procedure similar to MIMIC-EXTRACT (25). For the detection thresholds, we used thresholds found in Harutyunyan et al. (26). One set of upper and lower thresholds was used to filter outliers, and any value outside of this range was marked as missing. A set of thresholds were introduced to indicate the physiologically valid range. Any value that fell outside of this range was replaced with the nearest valid value, see Table 2.

**Table 2:** Summary of MIMIC-IV ED observation distribution of Vital Signs and ED LOS.

Variable	Total outliers	Total valid values	Valid range (26)
Triage temperature (°C)	23,903	401,178	[26, 45]
Triage heartrate (bpm)	17,097	407,984	[0, 350]
Triage resprate (bpm)	20,356	404,725	[0, 300]
Triage O2sat (%)	20,637	404,438	[0, 100]
Triage SBP (mmHg)	18,307	406,774	[0, 375]
Triage DBP (mmHg)	19,486	405,595	[0, 375]
Triage pain	38,236	386,846	[0, 10]
Triage acuity	6,987	418,094	[1, 5]
ED temperature last (°C)	26,837	398,200	[26, 45]
ED heartrate last (bpm)	18,438	406,643	[0, 350]
ED resprate last (bpm)	18,923	406,158	[0, 300]
ED O2sat last (%)	29,102	395,979	[0, 100]
ED SBP last (mmHg)	18,687	406,403	[0, 375]
ED DBP last (mmHg)	18,936	406,145	[0, 375]
ED pain last	49,650	375,432	[0, 10]
ED LOS (h)	3,053	410,132	–

**Figure 2:**
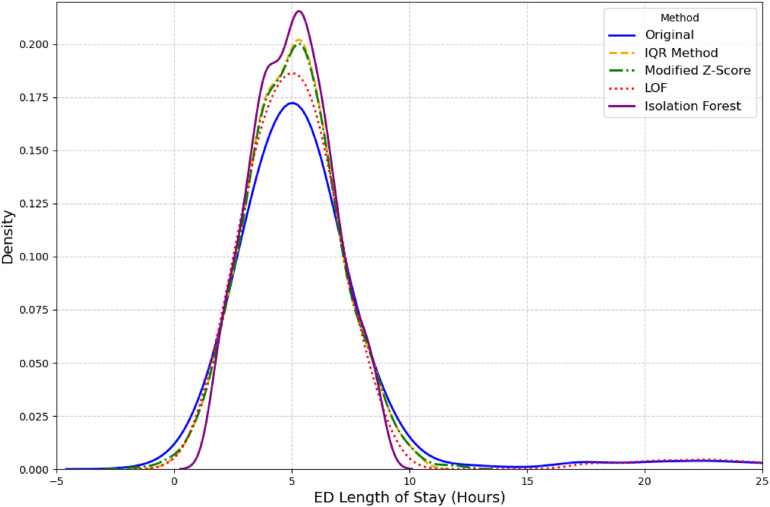
Comparison of ED length of stay distributions after outlier removal.

Second, outlier detection was performed on the outcome variable ED LOS. We used several established methods for this purpose, including the Interquartile Range (IQR), Modified Z-score, and unsupervised learning models like Isolation Forest and Local Outlier Factor (LOF) (27, 28). LOF outperformed other methods as it was able to retain a distribution closest to original distribution as seen in Figure 2. A complete overview of the total missing values for all variables discussed above can be found in Table 2.

#### 3.2.3 Outlier imputation experiment

Following the detection of outliers in both vital signs and ED Length of Stay (ED LOS) data, we conducted an extensive imputation experiment to benchmark various methods for handling missing values introduced by outlier detection. This experiment aimed to assess the accuracy of each imputation approach across these different types of clinical data.

##### 3.2.3.1 Imputation methods evaluated

The following imputation methods were applied to handle the artificially induced missing values for both vital signs and ED LOS data:

• **Median imputer**: The median value for each variable was used to replace missing values. This approach is commonly used for its robustness against outliers.
• **K-nearest neighbors (KNN) imputer**: The KNN imputer utilized the nearest five neighbors (n_neighbors = 5) to impute missing values. This method allows for local patterns in the data to be leveraged when estimating the missing values (29).
• **Iterative imputer**: A multivariate approach that iteratively estimated missing values using relationships between all other features. This method is particularly suited for datasets with highly correlated features (29).
• **Autoencoder imputer**: A deep learning-based autoencoder (30) was implemented . The model architecture consisted of an input layer matching the number of features, a dense hidden layer with 10 nodes using ReLU activation, and a reconstruction layer. Training was carried out using the dataset with missing values filled by median imputation, with 50 epochs and a batch size of 32.

##### 3.2.3.2 Evaluation metrics

To quantify the accuracy of each imputation method, two commonly used metrics were employed: Mean Squared Error (MSE) and Mean Absolute Error (MAE). These metrics compared the imputed values against the original values from the subset without missing data.

##### 3.2.3.3 Experimental design

The experiment was carried out separately for both the vital signs and the ED LOS variables. In each case, a large subset (*n* = 50,000/250,000) of relevant data without missing values was selected for evaluation. To simulate missing data, 20% of the values in the subset were randomly set to missing using a probabilistic mask. Then the various imputation methods were used to predict values and compare them with the original known value. This process was repeated fives times to ensure robustness in the evaluation and average MSE and MAE were found.

### 3.3 Data set

A master dataset of 410,927 Emergency Department (ED) visits from 202,503 unique adult patients (aged over 18) was generated, which included 72 different variables. The resulting dataset was a tabular dataset with a shape of (410,927 × 72), comprising both categorical and continuous features. Table 3 provides the baseline characteristics of the study population. The mean age of the patients in this dataset is 52.76 years, with a standard deviation of 20.61 years, indicating a broad age range ranging from 18 to 91 years. The racial makeup of our cohort offers crucial understanding of the study’s demographics, with primary groups being 53.81% White, 18.21% Black or African American, and 4.87% classified as Other. The data set’s gender breakdown shows a balanced representation, with a total count of 202,503 persons. 45.90% of these are men, while 54.10% are women. More information is provided in Table 3.

**Table 3:** Baseline characteristics of the study population.

	*n* = 410,927
Demographics	
Age (years)	52.76 (20.61)
Gender, *n* (%)	
Female	222,318 (54.10%)
Male	188,699 (45.90%)
Charlson comorbidity index score	1.37 (2.63)
Elixhauser comorbidity index score	3.09 (4.79)
Healthcare utilization	
Number of ED visits in past 30 d	0.24 (0.78)
Number of ED visits in past 90 d	0.53 (1.61)
Number of ED visits in past 365 d	1.40 (4.21)
Number of Hospital visits in past 30 d	0.16 (0.52)
Number of Hospital visits in past 90 d	0.36 (1.03)
Number of Hospital visits in past 365 d	0.97 (2.70)
Number of ICU visits in past 30 d	0.02 (0.16)
Number of ICU visits in past 90 d	0.05 (0.27)
Number of ICU visits in past 365 d	0.11 (0.50)
Medication administered during the previous visit, *n* (%)	
Yes	297,075 (72.29%)
No	113,852 (27.71%)
Triage vital signs	
Temperature (°C)	36.72 (0.54)
Heart rate (bpm)	85.22 (17.49)
Respiratory rate (bpm)	17.58 (2.50)
Oxygen saturation (%)	98.36 (2.40)
Systolic blood pressure (mmHg)	134.78 (22.13)
Diastolic blood pressure (mmHg)	77.39 (14.69)
Pain scale	4.11 (3.62)
Acuity	2.63 (0.71)
ED last vital signs	
Temperature (°C)	36.76 (0.37)
Heart rate (bpm)	78.19 (14.45)
Respiratory rate (bpm)	17.26 (2.48)
Oxygen saturation (%)	98.16 (2.93)
Systolic blood pressure (mmHg)	127.38 (19.56)
Diastolic blood pressure (mmHg)	73.55 (13.60)
Pain scale	2.18 (2.82)
Outcomes	
Length of stay (h), *n* (%)	
Short (≤ 4.5 h)	158,898 (38.67%)
Long (>4.5 h)	252,029 (61.33%)
Hospitalization, *n* (%)	
Yes	191,289 (46.55%)
No	219,638 (53.45%)
Critical, *n* (%)	
Yes	27,989 (6.81%)
No	382,938 (93.19%)
ED revisit 3 d, *n* (%)	
Yes	14,557 (3.54%)
No	396,370 (96.46%)

Continous variables presented as mean (sd) and categorical ones presented as count (percentage).

The outcome variable ED LOS was classified into two categories: short (less than 4.5 h) and long (greater than 4.5 h). This cutoff was chosen because it represents the average time hospitalized patients spend in the ED (22). According to relevant literature (31), the 4.5 h threshold aligns with several key performance metrics used in emergency care, which often consider a 4- to 5 h mark critical to evaluate quality of care and patient performance. The United Kingdom Department of Health (32) also supports the implementation of the 4 h rule. The outcome variable ED LOS had a mean value of 6.40, with a right-skewed distribution, as illustrated in Figure 2.

Initially, the data set was divided into training and test sets using a 80/20 ratio as detailed in Table 4. This method guarantees that the evaluation metrics accurately represent the model performance on unseen data.

**Table 4:** Comparison of ED LOS dataset distribution before and after SMOTETomek application.

Outcome: ED LOS		
	Short (≤4.5 h)	Long (>4.5 h)
Original data set (Imbalanced)		
Training data	127,168 (38.68%)	201,574 (61.32%)
Test data	31,726 (38.60%)	50,459 (61.40%)
Total	158,894 (38.67%)	252,033 (61.33%)
Balanced data set (SMOTETomek)		
Training data	201,489 (50.00%)	201,489 (50.00%)
Test data	31,726 (38.60%)	50,459 (61.40%)
Total	233,315 (48.07%)	251,948 (51.93%)

### 3.4 Data balancing methods for ED LOS

In this study, the ED Length of Stay (ED LOS) was identified as an imbalanced outcome variable, with the majority of instances classified as long stays (62%). To address this imbalance, we applied several data balancing techniques using the imblearn library in Python (33), focusing solely on the outcome variable ED LOS.

#### 3.4.1 Balancing methods

The following balancing methods were applied:

• **SMOTE (Synthetic minority over-sampling technique)**: The Synthetic Minority Over-sampling Technique (SMOTE) (34) addresses imbalanced data by creating synthetic samples for the minority class based on nearest neighbors. This increases minority representation and contributes to a more balanced training set.
• **ADASYN (Adaptive synthetic sampling)**: Adaptive Synthetic (ADASYN) (35) algorithm is similar to SMOTE, but it focuses on difficult-to-learn instances by creating synthetic samples in areas where minority classes are underrepresented.
• **Tomek links**: Tomek Links (36) is an undersampling method that eliminates overlapping samples from different classes, thereby improving class separation and lowering ambiguity.
• **SMOTETomek**: The SMOTE-Tomek method (12) combines SMOTE and Tomek Links to balance classes and remove overlapping samples, resulting in a better-defined dataset and improved classifier performance.
• **SMOTEENN**: The SMOTE-ENN method (37) combines SMOTE oversampling with Edited Nearest Neighbors (ENN) undersampling to generate synthetic minority samples and remove noisy or misclassified samples, improving data quality.

#### 3.4.2 Evaluation of balancing techniques

We evaluated the balanced datasets using various machine learning models, including Random Forest, Gradient Boosting, Logistic Regression, and a Multi-Layer Perceptron (MLP) neural network. A 5-fold stratified cross-validation was used to ensure the reliability of the metrics. Evaluation metrics were calculated for both the training and the test sets.

#### 3.4.3 Confusion matrix analysis

**Figure 3:**
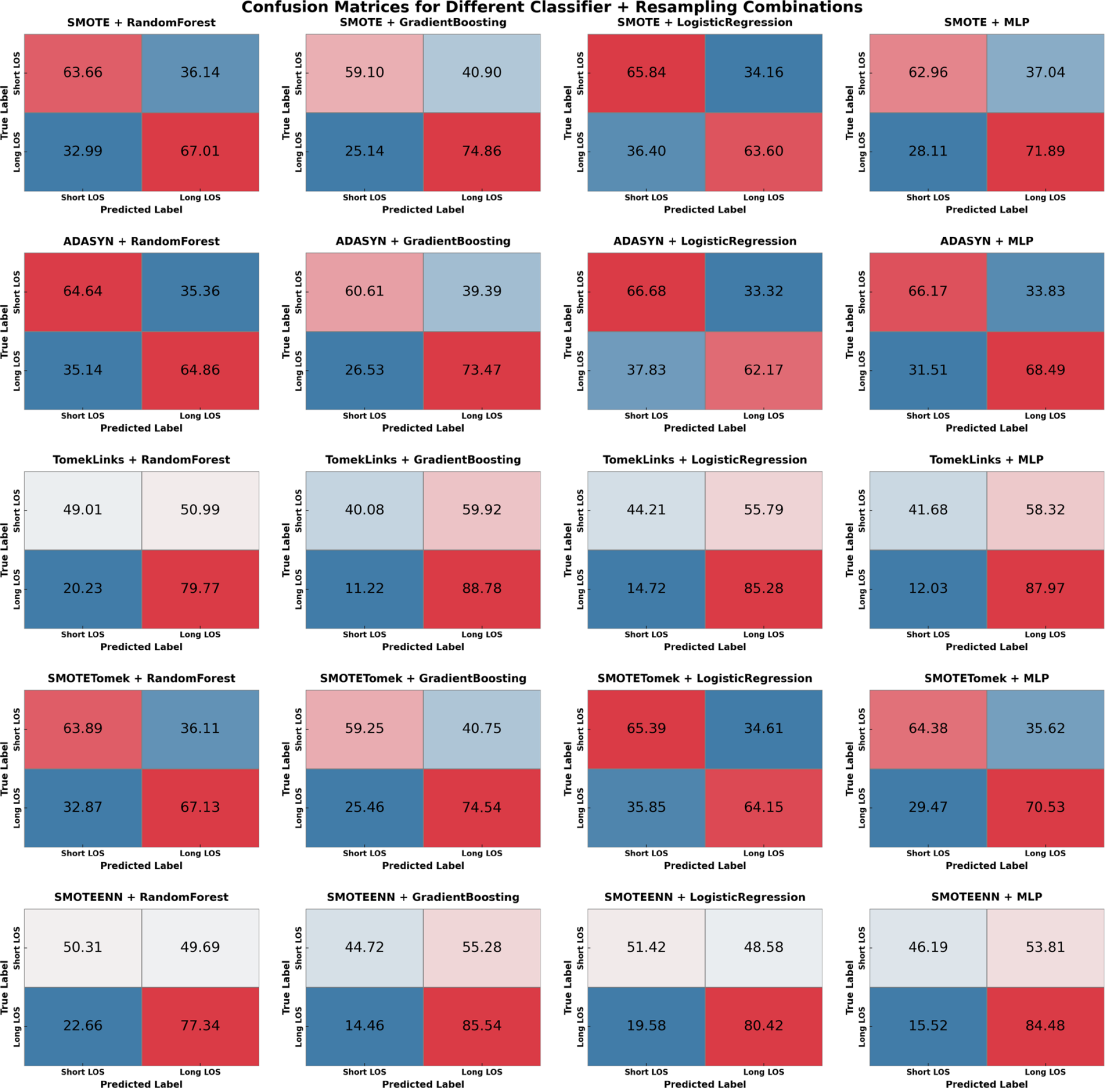
Confusion matrices for various sampling methods & classifiers. Where TP is ED LOS long stay and TN is ED LOS short stay. Values given in percentage format.

To better understand how data balancing affects model performance, we created a confusion matrix for each balancing technique and classifier. The confusion matrix provided a detailed view of true positives, true negatives, false positives, and false negatives, allowing us to evaluate the accuracy of each balancing strategy in correctly identifying both “Short” and “Long” ED LOS categories. Refer to Figure 3.

#### 3.4.4 Experimental procedure

The balancing and evaluation procedure consisted of the following steps:

1. **Splitting the dataset**: The dataset was divided into training and testing sets in an 80/20 split. To provide an unbiased evaluation of model performance, the training set was balanced while the test set remained unchanged.
2. **Cross-validation and resampling**: During the 5-fold stratified cross-validation, each training fold was resampled using one of the balancing techniques. The balanced training set was then utilized to train the models and validated on the corresponding unbalanced test fold to calculate accuracy, AUC, specificity, sensitivity, and F1 score.

### 3.5 Feature engineering and selection

#### 3.5.1 Feature engineering

In addition to the 72 analytical variables, several new variables were derived from the original data to improve the performance of the model. Notably, a new feature, *Medication Event*, was introduced as a boolean variable that indicates whether the patient received medication during a previous visit. The Charlson comorbidity index (CCI) and Elixhauser comorbidity index (ECI) were also calculated. For both indices, weights ranging from 1 to 6 were assigned to various comorbid conditions based on their severity (38). These weights were then summed together to generate a single score for each index, providing a comprehensive measure of each patient’s comorbidity burden.

To facilitate prediction of the outcome, the length of stay (“ED_LOS”) variable was divided into two categories. Specifically, a 4.5 h threshold was used to classify LOS as “short” (≤4.5 h) or “long” (>4.5 h). This classification produced a new binary variable in which short stays were encoded as 0 and long stays as 1, ensuring consistency for subsequent modeling, yielding 158,898 short stays (38.67%) and 252,029 long stays (61.33%).

#### 3.5.2 Encoding categorical features

To allow for more effective modeling, categorical variables were encoded using one-hot encoding utilizing the pandas library (39). This transformation enabled categorical variables to be represented by binary columns, providing flexibility for models that benefit from binary feature representations. Features encoded include *Race, Arrival Transport, Disposition, Insurance*, and *Chief Complaint* indicators. Each category within these characteristics was represented as a separate binary column to ensure completeness and preservation of information. Table S1 of the Supplementary Material provides a complete summary of the one-hot encodings.

#### 3.5.3 Feature selection

In order to perform feature selection, a comprehensive feature importance analysis was performed. This process involved taking advantage of the inherent ability of the Gradient Boosting (GB) model to rank features based on their predictive power (40). By analyzing the importance scores of the GB model, we identified the ten most influential variables for ED LOS. This step was critical in reducing the dimensionality, which simplified the data and increased the efficiency of subsequent analysis. By limiting the variables to those that were the most important, we not only improved the interpretability of our model, but also reduced computational complexity and the risk of over-fitting (41). Importance scores provided by the GB model highlighted key variables such as patient demographics, initial triage vital signs, and comorbidities that significantly affect ED LOS. The list of extracted variables and their description is provided in Table 5.

**Table 5:** Description of top 10 GB importance variables.

Variable name	Description	Type
Triage acuity	Triage acuity [emergency severity index (1: Resuscitation/2: Emergent/3: Urgent/4: Less urgent/5: Non-urgent)]	Categorical
Arrival transport unknown	Indicator for unknown mode of patient arrival to the ED (YES/NO).	Boolean
Elixhauser comorbidity index (ECI)	Elixhauser Comorbidity Index (ECI) score, representing the comorbidity burden of the patient.	Numeric
Disposition admitted	Indicator for whether the patient was admitted to the hospital from the ED (YES/NO).	Boolean
Chief complaint abdominal pain	Indicator for whether the chief complaint at triage was abdominal pain (YES/NO).	Boolean
Arrival transport ambulance	Indicator for whether the patient arrived at the ED via ambulance (YES/NO).	Boolean
Age	Age of the patient.	Numeric
Medication event	Indicator for whether the patient was given medication in a prior visit (YES/NO).	Boolean
Disposition home	Indicator for whether the patient was discharged home from the ED (YES/NO).	Boolean
Disposition left without being seen	Indicator for whether the patient left the ED without being seen by a clinician (YES/NO).	Boolean

### 3.6 Machine learning and evaluation methods

#### 3.6.1 Machine learning methods

Following the successful data preprocessing and feature selection, we evaluated four machine learning methods: Gradient Boosting (GB), Random Forest (RF), Logistic Regression (LR), and a Multi-Layer Perceptron (MLP) neural network. We used the scikit-learn package (42) for tree-based methods and logistic regression, and Keras (43) to implement the MLP model.

The MLP architecture, consisting of an input layer of 10 neurons and a single hidden layer of 64 neurons with ReLU activation as seen in Supplementary Figure S1, was inspired by previous work in healthcare prediction tasks (7). The hyperparameters and model parameters used for each machine learning method, and MLP, are summarized in Table 6.

**Table 6:** Machine learning modeling parameters.

Algorithm	Parameter	Default value
Logistic regression	Maximum iterations	1000
Random forest	No. of estimators	100
Gradient boosting	No. of estimators	100
Multi-layer perceptron	Learning rate	0.001
	Batch size	200
	Epochs	20

#### 3.6.2 Evaluation methods

To thoroughly assess the performance of our prediction models, we examined the receiver operating characteristic (ROC) curve and reported the area under the curve (AUC) as the primary indicator of overall predictive performance. Furthermore, additional evaluation metrics such as accuracy, sensitivity, specificity, and F1 score were calculated during model training and testing to gain a comprehensive understanding of each model’s effectiveness.

In addition, to ensure robustness, we used five-fold stratified cross-validation during model training. This technique ensures that each fold has a similar class distribution, providing a more reliable evaluation of the model performance (44).

Furthermore, we used McNemar’s test (45) to compare the performance of the models pairwise. McNemar’s test, a non-parametric statistical test, was used to see if the differences in the misclassification rates between models were statistically significant. The contingency tables were created using the model predictions, and p-values less than 0.05 were used to indicate significant differences in model performance.

### 3.7 Rule extraction

The purpose of extracting rules from the ensemble techniques is to make the model decision logic clearer and more transparent to domain experts while retaining the predictive power and precision that the ensemble methods offer (46). In this paper, we used TE2Rules (47) rule extraction algorithm which has four primary stages:

• At first, TE2Rules generates initial rule candidates by associating rules with distinct TE tree nodes. These rules describe the decision paths that lead from the root to each node.
• The second stage assesses these candidates based on two criteria: the presence of positive instances covered by the rule and rule precision greater than a predefined threshold. Rules that do not meet these requirements are eliminated.
• In the third stage, TE2Rules develop stricter rule candidates by merging pairs of existing candidates. This process is repeated in phases, eventually refining the rules. Importantly, TE2Rules often converges to a high level of fidelity in a few stages, minimizing the requirement for in-depth processing.
• Finally, once TE2Rules has identified rules that cover all positive cases, it reduces the rule list by identifying the smallest subset of rules that cover all positives collectively. This phase minimizes redundancy and improves the interpretability of the rules, making it helpful for understanding the Tree Ensemble model’s decision-making process (47).

### 3.8 Rules analysis

In order to substantiate the reliability and effectiveness of the rules derived from the Gradient Boosting model, we performed a comprehensive analysis of the rules by extracting and evaluating three key metrics. The generated metrics include overall coverage, relative coverage, and rule accuracy, each of which is crucial to evaluate the practical utility and precision of the rules.

#### 3.8.1 Overall coverage

Overall coverage quantifies the proportion of the data set that is covered by a given rule (10). It indicates how extensively a rule applies across the entire dataset. A higher coverage suggests that the rule is applicable to a larger segment of the data, which can be indicative of its general relevance.

$$\text{Overall coverage}=\left(\frac{\text{Number of samples where rule is activated}}{\text{Total number of samples}}\right)\times 100$$

#### 3.8.2 Relative coverage

Relative coverage measures the proportion of samples in a specific category that are covered by the rule (48). This metric helps us understand how well the rule performs for a particular category such as long or short ED stays, reflecting the rule’s effectiveness in distinguishing between different categories within the dataset.

$$\text{Relative coverage}=\left(\frac{\begin{array}{c} \text{Number of samples where rule is activated}\\ \text{and belongs to category ( ED LOS ( short/long) ) } \end{array}}{\text{Total number of samples}}\right)\times 100$$

#### 3.8.3 Rule accuracy

Rule Accuracy measures the proportion of correctly classified samples out of all samples where the rule is activated (10). This metric evaluates how accurately the rule identifies the intended category and misclassified. Greater accuracy demonstrates the rule’s effectiveness in producing correct predictions upon activation.

$$\text{Rule accuracy}=\left(\frac{\text{Number of correctly classified samples}}{\text{Number of activated samples}}\right)\times 100$$

## 4 Results

In this section, we present the findings of our investigations. We begin by presenting our experimental results, feature analysis to gain a better understanding of the data set. Next, we showcase the benchmark task’s models and derived rules.

### 4.1 Imputation experiment results

To evaluate the effectiveness of different imputation methods, we performed an extensive imputation experiment. Table 7 summarizes the average results from the experiments. Notably, the Iterative Imputer performed the best, with the lowest average MSE and MAE across both sample sizes (50,000 and 250,000) and was used for replacing missing values earlier detected. The KNN Imputer and Median Imputer performed similarly but slightly underperformed compared to the Iterative Imputer. Despite its complexity, the Autoencoder Imputer had a higher error rate, indicating that its performance may not be as generalizable for this dataset.

**Table 7:** Average performance of imputation methods.

Sample size	Imputation method	Average MSE	Average MAE
50,000	Median imputer	24.89	1.23
	KNN imputer	26.38	1.27
	Iterative imputer	**16.45**	**0.98**
	Autoencoder imputer	32.10	2.79
250,000	Median imputer	24.83	1.23
	KNN imputer	27.23	1.29
	Iterative imputer	**16.44**	**0.98**
	Autoencoder imputer	26.68	1.85

Boldface denotes lowest average MSE/MAE.

### 4.2 Balancing datasets experiment results

The evaluation of various sampling methods combined with machine learning models to predict ED LOS yielded insightful findings. The sampling techniques employed were ADASYN, SMOTE, SMOTEENN, SMOTETomek, and TomekLinks. The performance metrics for testing and training–AUC, Accuracy, F1 Score, Sensitivity, and Specificity–are detailed in Supplementary Materials S2, S3, with confusion matrices available in Figure 3

SMOTETomek emerged as the most balanced method across all metrics. SMOTETomek consistently achieved high Area Under the Curve (AUC) scores across models, with the MLP model having the highest AUC of 0.731, followed by Gradient Boosting at 0.729. SMOTETomek’s specificity was also consistently high across multiple models, achieving 65.65% with LR, demonstrating its ability to accurately identify patients predicted to have a short ED LOS. The confusion matrix for SMOTETomek combined with MLP also advocated for its balanced performance, with 64.38% of short LOS and 70.53% of long LOS correctly predicted, indicating that SMOTETomek could maintain a well-distributed prediction across both classes while maintaining relatively low FPs and FNs.

The analysis of the confusion matrices (Figure 3) provides additional support for these findings. SMOTETomek combined with GB produced a well-balanced prediction result, with 59.10% of the short LOS cases correctly identified and 74.86% of the long LOS cases correctly predicted. Similarly, SMOTETomek with LR correctly identified 64.15% of long LOS cases and predicted short LOS with 65.39% accuracy, achieving higher specificity than sensitivity in this case. Another notable result was TomekLinks combined with GB, which correctly predicted 88.78% of long LOS cases, the highest among all models and sampling methods, demonstrating its efficacy in detecting patients who may require prolonged hospital stays. TomekLinks + MLP also had a high true positive rate of 87.97%, demonstrating its utility in accurately detecting long stays. In contrast, ADASYN outperformed all other classifiers in terms of specificity. Specifically, ADASYN combined with Logistic Regression achieved the highest specificity of 66.68% among all methods and classifiers, while ADASYN combined with Gradient Boosting demonstrated a strong AUC of 0.6998 and a specificity of 60.61%. However, this came at the expense of sensitivity, which was consistently lower than in other sampling methods.

Logistic regression, when combined with SMOTE, ADASYN, and SMOTETomek, consistently achieves greater specificity than sensitivity, as shown in Table S2. This distinct pattern, which does not appear with other classifiers, suggests that LR is effective in distinguishing Short ED LOS while maintaining a reasonably balanced recall for Long ED LOS. For example, SMOTE with LR correctly predicts 65.84% of short ED LOS cases compared to 63.60% to long ED LOS. Specificity outperformed sensitivity for the mentioned sampling methods, indicating that the LR classifier was better at correctly predicting Short ED LOS than Long ED LOS.

Overall, SMOTETomek appears to provide the most balanced performance, with high AUC, Sensitivity, and relatively high Specificity across multiple classifiers. Although TomekLinks had the highest sensitivity, it tended to sacrifice Specificity, making SMOTETomek the more robust option when considering overall balance. Other methods, such as SMOTEENN and ADSYN, also performed well, particularly in maintaining a high F1 score, which may be appropriate when balancing precision and recall is the main goal. This balancing experiment ensured that the models developed to predict ED LOS could learn equally from both categories, resulting in better and more actionable clinical insights.

### 4.3 Feature analysis

Our analysis provides critical information on the factors that influence ED LOS. It contains detailed information about patient characteristics, including a broad age range and various influencing variables. Table 8 shows key variables related to ED LOS classified as short (≤4.5 h) and long (>4.5 h), along with their Gradient Boosting (GB) importance scores and *t* statistics where continuous variables are presented as mean (sd), while categorical variables are presented as count (percentage). All variables were found to be statistically significant with a p-value <0.01, as indicated by t statistics, underscoring their influence on ED LOS.

**Table 8:** Summary statistics and gradient boosting (GB) importance for ED LOS outcomes.

Variable name	Outcome		*T*-Statistic	GB importance
	ED LOS			
	Short (≤4.5 h)	Long (>4.5 h)		
Triage acuity	2.78 (0.79)	2.53 (0.63)	109.311 (S)	0.397
Disposition admitted			−116.812 (S)	0.118
YES	42,837 (26.96%)	112,957 (44.82%)		
NO	116,061 (73.04%)	139,072 (55.18%)		
Elixhauser comorbidity index score (ECI)	2.10 (4.06)	3.73 (5.10)	−107.314 (S)	0.102
Arrival transport unknown			90.953 (S)	0.101
YES	18,316 (6.49%)	3,334 (1.32%)		
NO	148,582 (93.51%)	248,695 (98.68%)		
Chief complaint abdominal pain			−81.656 (S)	0.077
YES	10,212 (6.43%)	37,063 (14.71%)		
NO	148,686 (93.57%)	214,966 (85.29%)		
Arrival by ambulance			−98.076 (S)	0.061
YES	43,313 (27.26%)	106,363 (42.20%)		
NO	115,585 (72.74%)	145,666 (57.80%)		
Age	48.19 (20.47)	55.65 (20.17)	−114.811 (S)	0.053
Medication administered during previous visit			−105.107 (S)	0.040
YES	100,382 (63.17%)	199,693 (78.04%)		
NO	58,516 (36.83%)	55,336 (21.96%)		
Disposition left without being seen			83.000 (S)	0.033
YES	5,092 (3.20%)	427 (0.17%)		
NO	153,806 (96.80%)	251,602 (99.83%)		
Disposition home			94.958 (S)	0.018
YES	104,881 (66.01%)	128,794 (51.10%)		
NO	54,017 (33.99%)	123,325 (48.90%)		

All variables were found to be statistically different (S) with p-value < 0.01.

The t statistic indicates significant differences between the short and long stay groups in all variables. The mean triage acuity scores are slightly higher for patients with short stays (2.78) than long stays (2.53), with a high GB importance score of 0.397. 6.49% of the patients with a short stay arrived by unknown means of transport, compared to 1.32% of the long-term patients, with an importance score of 0.083. The Elixhauser Comorbidity Index Score averages 2.10 for shorter stays and 3.75 for longer stays, with an importance score of 0.102. Furthermore, 44.82% of the long-term patients were later admitted, compared to 26.96% of the patients with a short-term stay, with an importance score of 0.118. Moreover, 27.26% of patients who had a short stay arrived by ambulance, whereas 42.20% of long stay patients arrived by ambulance, with an importance score of 0.061.

Additionally, 63.17% of patients who had a short stay in the ED were recorded to have received medication during a previous visit, compared to 78.04% of those with a long stay, with an importance score of 0.040. Moreover, 66.01% of the patients with a short stay were discharged home, while 51.10% of patients with a long stay experienced the same result, whereas 3.20% of patients with short stays left without being seen, compared to 0.17% of patients with long stays. The importance scores for these are 0.018 and 0.033, respectively.

### 4.4 Model evaluation

The top 10 previously identified variables were used to categorize ED LOS. Table 4 shows the distribution of training and test data based on ED LOS outcomes. In the original unbalanced data set, the training data have 61.32% long stays and 38.68% short stays, while the test data have 61.40% long stays and 38.60% short stays. After using SMOTETomek, the training set has a perfectly balanced distribution of 50.00% long stays and 50.00% short stays. In general, the data set had a distribution of 61.33% long stays and 38.67% short stays in the original data, vs. 51.93% long stays and 48.07% short stays in the balanced data set of SMOTETomek.

Next, we evaluated various machine learning models for predicting ED LOS using both the original unbalanced data set and a balanced version generated by SMOTETomek. The evaluation uses metrics such as accuracy, sensitivity, specificity, AUC score, and F1 score, as shown in Table 9.

**Table 9:** Average of 5-folds model performance on original and balanced datasets.

Model	Accuracy	Sensitivity	Specificity	AUC score	F1 score
Original data set (average training scores ± S.D)					
Gradient boosting	69.98% ± 0.0006	**88.24%** ± 0.005	41.01% ± 0.009	0.731 ± 0.0007	0.783 ± 0.0006
Random forest	**73.29%** ± 0.03	83.67% ± 0.002	**56.82%** ± 0.003	**0.805** ± 0.0004	**0.794** ± 0.0005
Logistic regression	69.30% ± 0.0002	85.81% ± 0.0008	43.12% ± 0.001	0.699 ± 0.0002	0.774 ± 0.0002
MLP	69.75% ± 0.002	83.59% ± 0.029	47.80% ± 0.042	0.730 ± 0.001	0.772 ± 0.007
Original data set (average test scores ± S.D)					
Gradient boosting	69.93% ± 0.001	**88.20%** ± 0.005	40.95% ± 0.008	**0.730** ± 0.002	**0.782** ± 0.001
Random forest	67.99% ± 0.002	79.26% ± 0.002	**50.11%** ± 0.007	0.701 ± 0.0016	0.752 ± 0.0008
Logistic regression	69.29% ± 0.0008	85.81% ± 0.0009	43.10% ± 0.003	0.699 ± 0.001	0.774 ± 0.0004
MLP	**69.71%** ± 0.003	83.51% ± 0.029	47.82% ± 0.040	**0.730** ± 0.002	0.772 ± 0.008
Balanced data set (SMOTETomek) (average training scores ± S.D)					
Gradient boosting	68.89% ± 0.001	**75.39%** ± 0.004	58.59% ± 0.004	0.730 ± 0.0006	0.748 ± 0.002
Random forest	**71.54%** ± 0.0005	71.58% ± 0.001	**71.48%** ± 0.001	**0.800** ± 0.002	**0.755** ± 0.0005
Logistic regression	64.61% ± 0.002	63.99% ± 0.005	65.59% ± 0.004	0.699 ± 0.0001	0.689 ± 0.003
MLP	68.55% ± 0.002	72.89% ± 0.013	61.66% ± 0.015	0.732 ± 0.0011	0.740 ± 0.005
Balanced data set (SMOTETomek) (average test scores ± S.D)					
Gradient boosting	**68.86% ± 0.002**	**75.36% ± 0.002**	58.55% ± 0.003	0.729 ± 0.002	**0.748 ± 0.002**
Random forest	65.43% ± 0.0003	66.61% ± 0.0014	63.55% ± 0.002	0.700 ± 0.002	0.703 ± 0.0005
Logistic regression	64.64% ± 0.001	64.00% ± 0.004	**65.65% ± 0.004**	0.699 ± 0.001	0.689 ± 0.003
MLP	68.53% ± 0.003	72.90% ± 0.013	61.60% ± 0.013	**0.731 ± 0.001**	0.740 ± 0.005

Boldface denotes best performing model with respect to metric.

GB performed the best on the unbalanced dataset, with an AUC score of 0.730, F1 score of 0.782, accuracy of 69.93%, and sensitivity of 88.20%. This indicates that GB had strong predictive power, particularly in distinguishing between patients with long and short ED stays. However, the specificity for GB remained low at 40.95%, indicating a tendency to overestimate the majority class (long stays) at the expense of accurately predicting the minority class (short stays). RF also performed well, with an AUC of 0.701 and the highest specificity (50.11%) among the models, implying that it was more effective in correctly identifying patients with short stays than GB. MLP also did well obtaining the highest accuracy on the original dataset (69.71%). MLP performed similar to LR in terms of Specificity, but MLP slightly outperformed LR.

The use of SMOTETomek significantly altered the model dynamics. GB continued to outperform other models on the balanced dataset, with the highest accuracy (68.86%), sensitivity (75.36%), and F1 score (0.748). This suggests that GB was able to maintain its robustness despite the use of SMOTETomek. RF also performed well in the balanced context, achieving one of the highest specificity (63.55%) but with a notable trade-off in sensitivity. LR model improved its specificity to 65.65%, the highest among all models. However, sensitivity decreased, indicating an increased focus on correctly predicting short stays at the expense of long stays.

Training scores across models followed consistent trends with test scores, demonstrating the reliability of the evaluation metrics. For example, MLP had the joint highest training AUC (0.730) in the unbalanced dataset, which corresponded to its strong performance in specific metrics during testing. Interestingly, the F1 scores for GB and MLP remained relatively high in both the original and balanced datasets, demonstrating the models’ consistent ability to balance precision and recall effectively.

McNemar’s test was run on both the unbalanced and balanced datasets results to better understand the differences in model performance. The results, which are summarized in Supplementary Table S4, show that for the majority of comparisons, the differences in model performance were statistically significant, with the exception of GB vs MLP on the balanced dataset, where the difference was not significant. This implies that GB and MLP performed similarly on the balanced data, indicating that both models were equally capable of capturing the nuances of ED LOS predictions and the difference in performance was not statistically significant.

In addition, we conducted minority class analysis for the short stay. Although the labels were flipped and minority class was now “long stay,” the overall model performance has remained largely consistent, demonstrating robustness to change. The specificity had generally increased in comparison to the sensitivity, indicating a reversal of focus, with the models now correctly predicting the minority class (long stays) more frequently, at the expense of sensitivity. For example, Gradient Boosting (GB) had a specificity of 88.43% and a sensitivity of 40.76% for the minority class, indicating that it was more accurate in predicting “long stay” cases. Similarly, Random Forest (RF) showed 79.75% specificity and 49.22% sensitivity for the minority class. This is a significant but expected contrast to the previous evaluation, in which sensitivity was higher, indicating a shift in the models’ prediction tendencies. Furthermore, Logistic Regression (LR) and Multi-Layer Perceptron (MLP) demonstrated similar trade-offs, with both models having higher specificity than sensitivity, highlighting the consistent trend summarized in Supplementary Table S5.

### 4.5 Rules extracted

We used TE2Rules (47) to derive a global rule list from a 100-tree Gradient Boost Model, with a precision threshold of 90% set. Table 10 provides a detailed summary of the rules and fidelity metrics for predicting Emergency Department Length of Stay (ED LOS) for both long and short outcomes. Six rules for long stays explain 88.16% of the overall predictions, including all positive predictions and 48.30% of the negative predictions. The key variables in these rules include age, type of arrival transport, triage acuity, and chief complaint.

**Table 10:** Rules and metrics for short (≤4.5) and Long (>4.5 h) Outcomes. (see Table 12 for rule performance metrics).

Outcome	Details
Short stay (ED LOS ≤ 4.5 h)	Number of rules: 7
	Overall prediction explanation: 96.91%
	Positive prediction explanation: 93.99%
	Negative prediction explanation: 97.29%
Rules	Description
Short rule 1	Arrival transport ambulance = NO & chief complaint abdominal pain = NO
	& Elixhauser comorbidity index (ECI) score ≤ 0.5 & medication event = NO
	& Triage acuity = [urgent/less urgent/non-urgent]
Short rule 2	Age ≤ 30.5 & arrival transport ambulance = NO
	& Chief complaint abdominal pain = NO & triage acuity = [urgent/less urgent/non-urgent]
Short rule 3	Triage acuity = [less urgent/non-urgent]
Short rule 4	Arrival transport unknown = YES
Short rule 5	32.5<Age≤37.5 & Arrival transport ambulance = NO
	& Chief complaint abdominal pain = NO & elixhauser comorbidity index (ECI) score ≤ 0.5
	& Triage acuity = [urgent/less urgent/non-urgent]
Short rule 6	38.5<Age≤45.5 & arrival transport ambulance = NO
	& Arrival transport unknown = NO & chief complaint abdominal pain = NO
	& Disposition admitted = NO & elixhauser comorbidity index (ECI) score ≤ 0.5
	& Triage acuity = [urgent]
Short rule 7	Disposition left without being seen = YES
Long stay (ED LOS > 4.5 h)	Number of rules: 6
	Overall prediction explanation: 88.16%
	Positive prediction explanation: 100%
	Negative prediction explanation: 48.30%
Rules	Description
Long rule 1	Age > 29.5 & Arrival transport unknown = NO
	& Triage acuity = [resuscitation/emergent/urgent]
Long rule 2	Elixhauser comorbidity index (ECI) score > 0.5
Long rule 3	Arrival transport ambulance = YES
Long rule 4	Chief complaint abdominal pain = NO
	& Disposition home = NO & Disposition left without being seen = NO
Long rule 5	Chief complaint abdominal pain = YES
Long rule 6	Age ≤ 70.5 & Triage acuity = [resuscitation/emergent]

In contrast, the seven rules for short stays explain 96.91% of the overall predictions. These rules cover 93.99% positive predictions and 97.29% negative predictions. Factors such as arrival transport type, chief complaint of abdominal pain, and triage acuity also play significant roles in these rules. Each rule is detailed with specific conditions, allowing a clear view of the model’s decision boundaries for distinguishing between short and long ED LOS outcomes.

Furthermore, Table 11 summarizes the data observations related to the activation of a certain rule. Notably, there are no cases in which rules for both short and long stays are not activated, and a total of 49,364 observations show simultaneous activations for both short and long rules, indicating dilemma points.

**Table 11:** Summary of rule activation and stay categories.

Category	Short stay (≤4.5 h)	Long stay (>4.5 h)
No rules activated (short stay)	314,341	–
No rules activated (long stay)	–	47,222
No rules activated (both)	0	0
Dilemma points (both rules activated)	49,364	49,364

Moreover, Table 12 provides metrics for each rule, including coverage and accuracy. For long stay rules, rule 5 has the highest overall accuracy at 78.40% whereas long rule 1 has the highest overall coverage where it covers 74.78% of the data. Conversely, for short stay rules, rule 7 has the highest accuracy at 92.26%, despite having a lower overall coverage of 1.34%. This contrast highlights the varying efficacy of rules in predicting different outcomes. long stay rules have higher coverage but vary significantly in accuracy, with some rules achieving high coverage while having lower accuracy. Short stay rules, on the other hand, can achieve very high accuracy despite covering less of the dataset, indicating that they are more specialized in identifying true short stay cases.

**Table 12:** Rules empirical metrics (see Table 10 for rules).

Rule	Overall coverage (%)	Category	Relative coverage (%)	Accuracy (%)
Short rule 1	10.54	Short	7.30	69.27
		Long	3.24	30.73
Short rule 2	10.10	Short	6.77	66.97
		Long	3.34	33.03
Short rule 3	7.06	Short	5.74	81.30
		Long	1.32	18.70
Short rule 4	3.32	Short	2.51	75.58
		Long	0.81	24.42
Short rule 5	2.28	Short	1.42	62.23
		Long	0.86	37.77
Short rule 6	1.75	Short	0.97	55.33
		Long	0.78	44.67
Short rule 7	1.34	Short	1.24	92.26
		Long	0.10	7.74
Long rule 1	74.78	Short	23.49	31.42
		Long	51.28	68.58
Long rule 2	43.24	Short	12.43	28.75
		Long	30.81	71.25
Long rule 3	36.42	Short	10.54	28.94
		Long	25.88	71.06
Long rule 4	18.11	Short	4.61	25.46
		Long	13.50	74.54
Long rule 5	11.50	Short	2.49	21.60
		Long	9.02	78.40
Long rule 6	7.45	Short	2.77	37.21
		Long	4.68	62.79

## 5 Discussion

### 5.1 Summary of key findings

This section presents the key findings of the research extensively. we start by examining the most important variables of our analysis, moving towards model evaluation and finally rules analysis.

#### 5.1.1 Key variables influencing ED LOS

The study identified several critical variables that significantly influence the LOS of the ED, with the following the most impactful:

1. Triage acuity: Patients with higher acuity levels were found to have shorter LOS, likely due to prioritization in treatment and faster resolution of less serious conditions.
2. Elixhauser comorbidity index (ECI): Significantly higher ECI scores correlated with longer LOS, reflecting the increased complexity and resource demands associated with treating patients with multiple comorbidities.
3. Arrival methods: Patients who arrived via ambulance or other known transport means had longer LOS, indicative of the more severe nature of their conditions requiring extended medical attention. Whereas, patients arriving with unknown means of transport had a shorter stay in the ED.
4. Previous medication history: A history of medication during previous ED visits was a strong predictor of longer LOS, indicating that ongoing treatment needs significantly influences the duration of stay.

#### 5.1.2 Model performance and comparison

##### 5.1.2.1 Gradient boosting (GB) and Multi-layer perceptron (MLP)

Gradient Boosting (GB) and Multi-Layer Perceptron (MLP) outperformed all other models tested, with high AUC scores, sensitivity, and consistent reliability across multiple metrics. GB performed slightly better overall, with an AUC score of 0.730, accuracy of 69.93%, sensitivity of 88.20%, and specificity of 40.95%. This suggests that GB was particularly effective at correctly predicting long-stay cases, while its specificity was relatively higher, indicating a greater emphasis on avoiding false positives, which is critical in emergency department settings. TomekLinks sampling paired with GB achieved the highest test sensitivity of 75.36%, making this model combination particularly suitable for scenarios where identifying all patients needing long stays is critical. However, this comes at the expense of specificity, which leads to a higher number of false positives for short stays.

Similarly, MLP showcased competitive performance with an AUC of 0.730, accuracy of 69.71%, and sensitivity of 83.51% and specificty of 47.82%. Although it did not surpass GB, MLP’s ability to balance between sensitivity and specificity makes it a strong candidate for ED LOS classification. Confusion matrix analysis for SMOTETomek + MLP reveals a balanced trade-off between sensitivity and specificity, indicating that it can correctly classify both long and short stays. For example, 72.90% of long stays were correctly predicted, and the true negative rate was also relatively high, resulting in fewer unnecessary prolonged hospitalizations than TomekLinks.

##### 5.1.2.2 Random forest (RF)

The Random Forest (RF) model performed well, but fell short of GB and MLP in terms of overall metrics. With an AUC of 0.701 and a specificity of 50.11%, RF was more effective in identifying patients who needed a short stay, providing a relatively balanced trade-off between true positives and true negatives. However, the sensitivity of 79.26% remained relatively moderate, indicating a slight weakness in accurately predicting long stays compared to the other models. The confusion matrices show that RF benefited significantly from the use of SMOTETomek, resulting in a balanced classification for both the ED and LOS classes. The true negative rate was 63.55%, indicating improved specificity after resampling.

According to Bentéjac et al. (49), gradient boosting outperforms random forest in terms of generalization, especially when dealing with class imbalances. GB’s sequential nature allows for iterative error correction, which makes it better suited to the task of discriminating between short and extended ED stays (13, 17). This is consistent with our findings, in which GB outperformed RF in terms of accuracy and AUC. Gradient boosting has the advantage due to its focus on misclassified samples during training, allowing for more efficient adaptation to the minority class (49).

##### 5.1.2.3 Logistic regression (LR)

Logistic Regression (LR) served as a baseline model and showed high specificity, especially when combined with SMOTETomek and ADASYN. LR had a test accuracy of 69.71% and a sensitivity of 85.81% on the unbalanced dataset, indicating its ability to accurately predict long stays and identify high-risk patients. However, the model lacked specificity (43.10%), making it unsuitable for predicting all patients who may require short stays.

LR consistently demonstrated higher specificity than sensitivity across various sampling methods, which corresponds to its lower complexity and linear decision boundary, effectively distinguishing short stays but missing long stays. The confusion matrices show that LR’s performance on the sampled data is characterized by a higher true negative rate, demonstrating its ability to prioritize minimizing false positives. The ADASYN + LR combination had the highest specificity of 66.68%, highlighting its utility in situations where reducing unnecessary admissions(short stays) is a priority.

#### 5.1.3 Confusion matrix analysis and practical implications

The analysis of confusion matrices reveals the various trade-offs each model makes between sensitivity and specificity. SMOTETomek emerges as a robust balancing method, especially when combined with MLP and GB, ensuring that long stays are properly identified while not jeopardizing short stay detection. This makes SMOTETomek suitable for clinical applications in which it is critical to identify high-risk patients while maintaining resource efficiency.

TomekLinks, when combined with GB or MLP, produced high sensitivity rates, indicating that it is effective in identifying the majority of patients who require extended care. However, this resulted in an increased false positive rate, implying that it should be used when patient safety takes precedence over resource allocation. In contrast, ADASYN combined with LR or RF provided greater specificity, emphasizing its efficacy in situations where reducing unnecessary long stays is critical to managing emergency department capacity.

Finally, GB and MLP consistently outperformed in terms of balanced performance, with both demonstrating balanced superior metrics. The clinical goal should determine the model and sampling technique; for example, if the goal is to reduce missed high-risk cases, TomekLinks with GB or MLP would be ideal. On the other hand, SMOTETomek combined with GB or MLP remains the best option for balanced, general applicability, ensuring a moderate trade-off between sensitivity and specificity.

#### 5.1.4 Rule extraction and analysis

##### 5.1.4.1 Overview of rules

A key innovation in this study was the use of a rule extraction technique on the Gradient Boosting model, which was intended to turn complex model decisions into human-interpretable rules. The TE2Rules algorithm (47) extracted 13 rules (6 long stay and 7 short stay) that encapsulate significant patterns and relationships identified by the model. The rules were developed using key predictors such as triage acuity, age, arrival methods, and specific patient conditions, and were intended to provide actionable insights for emergency department decision-making.

The extracted rules serve two primary functions: improving model interpretability and increasing transparency in clinical decision-making. By translating a Gradient Boosting model’s decision boundaries into a set of simple rules, healthcare professionals can gain a better understanding of why certain decisions were made. For example, these rules can be used to determine which patients are likely to spend long or short periods of time in the emergency department (ED), assisting with resource allocation and prioritizing patient care.

The rules were evaluated using several key metrics to determine their utility and reliability, including Overall Coverage. Higher overall coverage indicates that a rule is widely applicable across the dataset, implying general relevance. Rule 1 (Long Stay) had 74.78% coverage, indicating it applies to a significant portion of patient data. The broad applicability of the rule makes it especially useful for general triage decision-making. In addition, the accuracy of the rule is defined as the percentage of correctly classified samples among all samples where the rule is activated. High rule accuracy indicates that the rule is very effective in predicting the correct outcome in those cases. For instance, Rule 7 (Short Stay) had an extremely high rule accuracy of 92.26%, underscoring its strong predictive power in the subset it covers.

##### 5.1.4.2 Extracted rules and their implications

The extracted rules revealed important patterns, for instance:

• Mid aged or older patients (>29.5) with critical triage acuity ratings [Resuscitation/Emergent/Urgent] and known arrival methods are likely to experience long stays (Rule 1, Long Stay). Such rules allow ED staff to preemptively allocate more resources to patients fitting these criteria.
• Conversely, specific scenarios like patients leaving without being seen, strongly correlated with shorter stays (Rule 7, Short Stay). This insight can be used to identify potential gaps in care, such as situations in which patients may leave due to excessive wait times.

The rules have significant practical implications, particularly in terms of increasing transparency and trust in clinical machine learning models (8, 9). The model supports and enhances clinical judgment by providing healthcare professionals with easily interpretable rules. These rules could help with triage decisions, predict resource demands, and ultimately, improve patient outcomes by allowing for targeted interventions.

### 5.2 Comparison with previous studies

In this section, we compare the results of our study with those of previous research in predicting ED LOS. This analysis highlights the contributions of our methodology, especially in terms of the performance, interpretability, and importance of the identified factors. The studies are summarized in Table 1.

#### 5.2.1 Overview of methodologies and models

Several studies have utilized machine learning approaches to predict ED LOS, employing a spectrum of models ranging from conventional statistical techniques to advanced deep learning frameworks. For instance, Pedro et al. (15) used a PRO-AGE scoring system combined with bootstrapping to predict LOS in older adults, achieving an AUC of 0.79. Ala et al. (13) focused their efforts on fast-tracking patients (<4 h) in the ED, using models such as MLP, CART, and SGB which achieved a an AUC of 0.752. In contrast, Zebin et al. (14) employed an auto-encoded deep neural network, achieving an accuracy of 77.7%.

In our study, similar to the work of Etu et al. (17), we applied GB models, which consistently surpassed other models in terms of Accuracy and AUC scores, demonstrating their robustness in predicting ED LOS. Additionally, unlike Rahman et al. (19), who used a decision tree model to predict ED LOS greater than 4 h with an accuracy of 84.94%, our approach also integrates various approaches to handle class imbalance and derives transparent rule metrics, improving generalizability and transparency of our models.

#### 5.2.2 Feature selection and importance

The variables used in previous studies vary, but often include vital signs, demographic information, and admission details. For example, Ala et al. (13) highlighted chief complaints, age, and vital signs as crucial predictors, which aligns with the features we selected. Similarly, Zebin et al. (14) and Bopche et al. (16) emphasized the importance of demographics and ICD-9 diagnosis codes (chronic diseases), which are also consistent with our findings. Furthermore, factors such as age, mode of transport and triage acuity were identified as key predictors, supporting the findings of other studies, such as Chang et al. (18), who also highlighted similar variables as important in the prediction of ED LOS.

#### 5.2.3 Comparison of model performance

Gradient Boosting (GB) and Deep Learning models are frequently reported as top performers (7, 13, 14, 16, 17, 20). As an illustration, Etu et al. (17). reported a GB model with an AUC of 0.93, outperforming other tree-based classifiers such as Decision Trees and Random Forest in both imbalanced and SMOTE-balanced datasets. Similarly, our study demonstrated that the GB models provided the highest accuracy and AUC scores, reinforcing their effectiveness in ED LOS classification.

However, unlike previous studies, our research goes beyond typical performance metrics by integrating a novel rule extraction process (46). This approach improves interpretability and provides transparent actionable insights for clinical decision making (11). This process translates the complex decision logic of the model into simple, human-interpretable rules. Each extracted rule was analyzed for its overall coverage, relative coverage, and rule accuracy, metrics that have not been previously implemented in the context of ED LOS prediction. Few studies, such as Rahman et al. (19) and Bopche et al. (16), have explored this avenue using the J48 decision tree algorithm and SHAP, respectively. Our work builds upon this by applying and evaluating human-readable rules that require minimal expert interpretation, using various metrics for comprehensive assessment.

#### 5.2.4 Comparison of explainability methods

In the field of ED LOS prediction, explainability methods such as SHAP (Shapley Additive Explanations) and LIME (Local Interpretable Model-agnostic Explanations) have been used to clarify machine learning model decisions (16, 50, 51). For example, SHAP has been used to interpret feature contributions in predicting in-hospital mortality, long-term LOS and sepsis onset, providing consistent explanations across models. Similarly, LIME has been used to provide local approximations of model behavior, aiding understanding of individual predictions.

Our study distinguishes itself by incorporating the TE2Rules algorithm for rule extraction from a Gradient Boosting model. TE2Rules, in contrast to SHAP and LIME, which focus on feature importance and local approximations, translates complex model decisions into human-readable rules (46). This approach not only enhances interpretability but also enables actionable decision-making through explicit decision rules that clinicians can directly apply (52, 53).

For instance, TE2Rules generates simple “if-then” rules that summarize the underlying patterns in the data, making them easy to understand for healthcare professionals. Compared to feature importance scores (SHAP) or localized surrogates (LIME), TE2Rules offers a distinct advantage in transforming black-box models into user friendly rule-based logic. This rule-based explanation can directly guide treatment strategies or triage decisions, eliminating the need for additional interpretation layers. Increased transparency also helps with clinical accountability, as decision rules can be validated against medical guidelines and expert judgment, fostering trust in AI-driven recommendations.

#### 5.2.5 Novel contributions

Our work provides a thoughtful combination of methodological advancements and practical innovations that set it apart from previous research in the field. We offer several novel contributions that primarily aim to improve the interpretability, robustness, and practical applicability of predictive models for ED LOS:

• **Explainable AI techniques**: We used the TE2Rules technique for rule extraction, which greatly improves the interpretability of ensemble methods such as Gradient Boosting. This technique improves healthcare practitioner’s understanding of the decision-making process and builds trust in the model. Unlike traditional approaches that focus solely on model performance metrics like AUC, sensitivity, and specificity, our analysis offers a more granular analysis by dissecting the performance of each individual rule. This not only improves the interpretability of the model, but also provides a practical framework for applying these rules in real-world scenarios (10, 48). To our knowledge, the use of TE2Rules in predicting ED LOS is novel, as previous research has primarily focused on other explainability methods (50). For instance, some studies have used SHAP to interpret models that predict ED LOS, emphasizing feature importance but lack the simplicity of rule-based explanations.
• **Use of limited, clinically relevant features**: Unlike many previous studies, which relied on a relatively large number of variables (15–19), we used only 10 readily available and clinically significant features. This not only improves model simplicity, but also makes it easier to implement in real-world clinical settings while maintaining model accuracy and utility.
• **Robust evaluation on balanced and imbalanced data**: We evaluated model performance on both balanced and real-world imbalanced datasets in an exhaustive manner to ensure the findings’ robustness and practical relevance.

### 5.3 Limitations and future directions

Although the study demonstrated the effectiveness of rule extraction and model interpretability, it is necessary to highlight several limitations and suggest future avenues.

This study relies on historical data from a single source, which could introduce biases into model predictions and restrict the generalizability of the results. The performance of the different methods used in this study may vary in different healthcare settings.

The application of balancing in most cases improved specificity at the expense of sensitivity, indicating that while models became better at identifying the minority class (short ED LOS), they were less effective at detecting the majority class (long ED LOS). This trade-off suggests that the some balancing method may not be suitable for all clinical contexts, particularly where high sensitivity is crucial (54).

Furthermore, some rules, such as Long Rule 1, may exhibit a high overall coverage but a lower relative coverage, indicating a relatively high rate of misclassifications within the predicted category. For instance, Long rule 1 has an overall coverage of 74.78%, but its relative coverage for short stays is 23.49% and for long stays 51.28%, suggesting that while the rule applies to a large portion of the entire dataset, it may sometimes not accurately distinguish between short and long stays.

In addition, an argument-based framework that integrates learning and reasoning (53) will be explored. In this framework, knowledge is represented through object-level arguments that involve constructing arguments that link a set of premises (conditions describing a scenario) to the argument’s claim (the desired outcome). Two types of arguments are generated: object-level and priority arguments. Object-level arguments are factual claims and may support contradictory assertions, leading to opposing arguments. However, priority arguments establish a local preference between these arguments, determining their relative strength and intensifying the conflict between them. This framework will be exploited by analyzing the conflict between long and short rules.

Future research will also investigate the application of these techniques in diverse healthcare settings, evaluate the generalizability of generated rules, and improve rule extraction methods to better handle more complex cases and models.

## 6 Conclusion

This study demonstrates a robust machine learning-based approach to predicting Emergency Department Length of Stay (ED LOS), with Gradient Boosting (GB) emerging as the best performer in our analysis. Key predictors for ED LOS included Triage Acuity, Elixhauser Comorbidity Index, Arrival Methods, and Patient Medication History. Our use of multiple data balancing techniques, including SMOTETomek, effectively addressed class imbalance, but a trade-off between sensitivity and specificity persisted across models.

This work made an important contribution by extracting rules from the GB model using the TE2Rules algorithm. This process enabled us to create interpretable rules to aid clinical decision-making. These rules capture important relationships, such as patient age, triage acuity, and arrival conditions, which improves model transparency. Our findings indicate that combining predictive accuracy and interpretability via rule extraction can improve resource management and decision-making in emergency departments.

While the models produced promising results, there is still room for improvement in achieving a better balance of performance metrics. Future efforts will center on improving rule extraction processes, increasing model generalizability, and ensuring adaptability across various healthcare settings.

## Data Availability

Publicly available datasets were analyzed in this study. This data can be found here: Johnson et al. (22). In order to aid reproducibility, the code used in this study is available at the following GitHub repository: https://github.com/waziz786/MIMIC-IV-ED–CODE.
