# Supplementary material for: Leveraging machine learning and rule extraction for enhanced transparency in emergency department length of stay prediction
Source: Front Digit Health. 2025 Feb 12;6:1498939. doi: 10.3389/fdgth.2024.1498939 (PMC11861435; doi:10.3389/fdgth.2024.1498939)
Supplement: Supplementary file 1 [file Datasheet1.pdf]

Supplementary Material

1 SUPPLEMENTARY TABLES AND FIGURES

This document contains supplementary tables and figures referred to in the main text. The figures and tables are labeled as "S1", "S2", etc., to differentiate them from those in the main text.

1.1 Figures

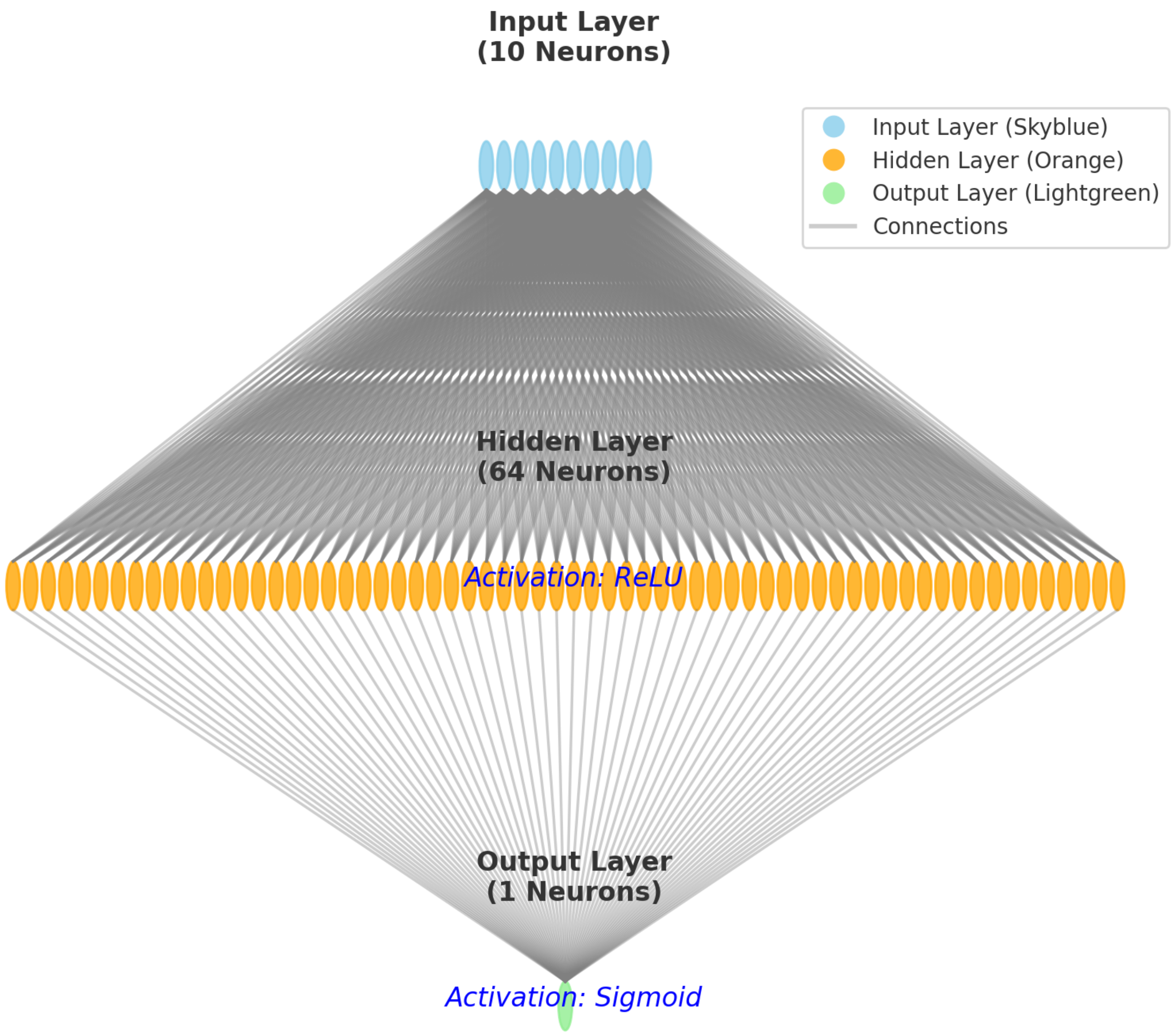

Figure S1: Multi-Layer Perceptron (MLP) Architecture

1.2 Tables

Table S1. One-Hot Encoding for Categorical Features

Feature	One-Hot Encoded Categories
**Race**	race_WHITE, race_BLACK/AFRICAN AMERICAN, race_OTHER, race_HISPANIC/LATINO - PUERTO RICAN, race_WHITE - OTHER EUROPEAN, race_HISPANIC/LATINO - DOMINICAN, etc.
**Arrival Transport**	arrival_WALK IN, arrival_AMBULANCE, arrival_UNKNOWN, arrival_OTHER, arrival_HELICOPTER
**Disposition**	disposition_HOME, disposition_ADMITTED, disposition_TRANSFER, disposition_LEFT WITHOUT BEING SEEN, disposition_ELOPED, etc.
**Insurance**	insurance_Unknown/NotInsured, insurance_Other, insurance_Medicare, insurance_Medicaid
**Chief Complaint**	chiefcom_chest_pain, chiefcom_abdominal_pain, chiefcom_headache, chiefcom_shortness_of_breath, chiefcom_back_pain, chiefcom_cough, chiefcom_nausea_vomiting, chiefcom_fever_chills, chiefcom_syncope, chiefcom_dizziness

Table S2. Test scores of sampling methods. Each value is highlighted to indicate the sampling method's rank for the classifier in terms of each metric. The colors represent rankings as follows: red (1st), blue (2nd), green (3rd), cyan (4th), black (5th).

	ADASYN	SMOTE	SMOTEENN	SMOTETomek	TomekLinks
**Test AUC**					
GradientBoosting	0.730	0.730	0.724	0.729	0.731
LogisticRegression	0.700	0.698	0.697	0.699	0.699
MLP	0.731	0.733	0.728	0.733	0.734
RandomForest	0.695	0.704	0.675	0.704	0.705
**Test Accuracy (%)**					
GradientBoosting	68.49	68.77	69.76	68.63	69.95
LogisticRegression	63.91	64.47	69.20	64.63	69.40
MLP	67.67	67.93	69.73	67.93	69.87
RandomForest	64.70	65.79	66.89	65.88	68.22
**Test F1 Score**					
GradientBoosting	0.741	0.746	0.776	0.745	0.784
LogisticRegression	0.679	0.687	0.762	0.6990	0.774
MLP	0.723	0.728	0.771	0.729	0.773
RandomForest	0.693	0.706	0.741	0.707	0.755
**Test Sensitivity (%)**					
GradientBoosting	73.47	74.86	85.54	74.54	88.78
LogisticRegression	62.17	63.60	80.42	64.15	85.28
MLP	68.85	69.85	82.95	70.19	83.47
RandomForest	64.86	67.01	77.34	67.13	79.77
**Test Specificity (%)**					
GradientBoosting	60.61	59.10	44.72	59.25	40.08
LogisticRegression	66.68	65.84	51.42	65.39	44.21
MLP	65.79	64.87	48.77	64.35	48.30
RandomForest	64.44	63.86	50.31	63.89	49.91

Table S3. Average Training scores of sampling methods. Each value is highlighted to indicate the sampling method's rank for the classifier in terms of each metric. The colors represent rankings as follows: red (1st), blue (2nd), green (3rd), cyan (4th), black (5th).

	ADASYN	SMOTE	SMOTEENN	SMOTETomek	TomekLinks
**Train AUC**					
GradientBoosting	0.728	0.729	0.722	0.729	0.730
LogisticRegression	0.699	0.699	0.697	0.699	0.699
MLP	0.731	0.731	0.722	0.732	0.733
RandomForest	0.690	0.700	0.674	0.700	0.700
**Train Accuracy (%)**					
GradientBoosting	68.50	68.82	69.69	68.86	69.93
LogisticRegression	64.32	64.52	69.01	64.63	69.21
MLP	68.19	67.70	69.66	68.23	69.92
RandomForest	64.18	65.40	66.65	65.43	67.98
**Train F1 Score**					
GradientBoosting	0.742	0.747	0.774	0.748	0.783
LogisticRegression	0.685	0.688	0.760	0.689	0.773
MLP	0.733	0.725	0.766	0.734	0.779
RandomForest	0.688	0.702	0.739	0.703	0.752
**Train Sensitivity (%)**					
GradientBoosting	73.84	75.19	84.82	75.35	88.43
LogisticRegression	63.38	63.67	80.13	64.00	85.67
MLP	71.25	69.40	81.16	71.34	86.54
RandomForest	64.35	66.55	77.07	66.61	79.26
**Train Specificity (%)**					
GradientBoosting	60.03	58.73	45.70	58.55	40.59
LogisticRegression	65.80	65.86	51.39	65.65	43.10
MLP	63.33	64.99	51.43	63.30	43.55
RandomForest	63.90	63.59	50.13	63.55	50.09

Table S4. McNemar Test Results for Model Comparisons on SMOTETomek Balanced and Original Unbalanced Datasets

	Chi-squared Statistic	p-value	Significance
**Unbalanced Dataset**			
RF vs GB	4101.0	3.67e-45	**S**
RF vs LR	5147.0	1.59e-18	**S**
RF vs MLP	3850.0	6.95e-50	**S**
GB vs LR	1812.0	2.35e-12	**S**
GB vs MLP	1370.0	0.495	**NS**
LR vs MLP	2559.0	9.81e-11	**S**
**Balanced Dataset**			
RF vs GB	4425.0	3.30e-134	**S**
RF vs LR	7893.0	1.24e-13	**S**
RF vs MLP	4005.0	5.89e-113	**S**
GB vs LR	4322.0	1.78e-234	**S**
GB vs MLP	2292.0	7.69e-07	**S**
LR vs MLP	3975.0	3.60e-209	**S**

Table S5. Minority Class Results for Model Evaluation (Training and Test Scores)

Model	Accuracy	Sensitivity	Specificity	AUC Score	F1 Score
**Average Training Scores**					
**Gradient Boosting**	70.01% $\pm$ 0.0004	40.77% $\pm$ 0.009	88.44% $\pm$ 0.006	0.731 $\pm$ 0.0003	0.513 $\pm$ 0.006
**Random Forest**	73.32% $\pm$ 0.0002	56.11% $\pm$ 0.004	84.17% $\pm$ 0.003	0.806 $\pm$ 0.0002	0.619 $\pm$ 0.001
**Logistic Regression**	69.36% $\pm$ 0.0003	43.37% $\pm$ 0.002	85.75% $\pm$ 0.0005	0.700 $\pm$ 0.0005	0.523 $\pm$ 0.0011
**MLP**	69.96% $\pm$ 0.0008	44.56% $\pm$ 0.017	85.97% $\pm$ 0.011	0.731 $\pm$ 0.0003	0.534 $\pm$ 0.0097
**Average Test Scores**					
**Gradient Boosting**	70.00% $\pm$ 0.0018	40.76% $\pm$ 0.01	88.43% $\pm$ 0.005	0.7308 $\pm$ 0.0018	0.5123 $\pm$ 0.0074
**Random Forest**	67.94% $\pm$ 0.0008	49.22% $\pm$ 0.003	79.75% $\pm$ 0.001	0.7022 $\pm$ 0.001	0.5428 $\pm$ 0.002
**Logistic Regression**	69.36% $\pm$ 0.0014	43.38% $\pm$ 0.0016	85.74% $\pm$ 0.0005	0.7000 $\pm$ 0.002	0.5227 $\pm$ 0.002
**MLP**	69.96% $\pm$ 0.002	44.54% $\pm$ 0.015	85.98% $\pm$ 0.011	0.7308 $\pm$ 0.002	0.5340 $\pm$ 0.008
